# Following the Script: How Drug Reps Make Friends and Influence Doctors

**DOI:** 10.1371/journal.pmed.0040150

**Published:** 2007-04-24

**Authors:** Adriane Fugh-Berman, Shahram Ahari

## Abstract

This article, which grew out of conversations between a former drug rep and a physician who researches pharmaceutical marketing, reveals the strategies used by reps to manipulate physician prescribing.

*It's my job to figure out what a physician's price is. For some it's dinner at the finest restaurants, for others it's enough convincing data to let them prescribe confidently and for others it's my attention and friendship...but at the most basic level, everything is for sale and everything is an exchange.*

—Shahram Ahari

*You are absolutely buying love.*

—James Reidy [1]

In 2000, pharmaceutical companies spent more than 15.7 billion dollars on promoting prescription drugs in the United States [2]. More than 4.8 billion dollars was spent on detailing, the one-on-one promotion of drugs to doctors by pharmaceutical sales representatives, commonly called drug reps. The average sales force expenditure for pharmaceutical companies is $875 million annually [3].

Unlike the door-to-door vendors of cosmetics and vacuum cleaners, drug reps do not sell their product directly to buyers. Consumers pay for prescription drugs, but physicians control access. Drug reps increase drug sales by influencing physicians, and they do so with finely titrated doses of friendship. This article, which grew out of conversations between a former drug rep (SA) and a physician who researches pharmaceutical marketing (AFB), reveals the strategies used by reps to manipulate physician prescribing.

## Better Than You Know Yourself

*During training, I was told, when you're out to dinner with a doctor, “The physician is eating with a friend. You are eating with a client.”*

—Shahram Ahari

Reps may be genuinely friendly, but they are not genuine friends. Drug reps are selected for their presentability and outgoing natures, and are trained to be observant, personable, and helpful. They are also trained to assess physicians' personalities, practice styles, and preferences, and to relay this information back to the company. Personal information may be more important than prescribing preferences. Reps ask for and remember details about a physician's family life, professional interests, and recreational pursuits. A photo on a desk presents an opportunity to inquire about family members and memorize whatever tidbits are offered (including names, birthdays, and interests); these are usually typed into a database after the encounter. Reps scour a doctor's office for objects—a tennis racquet, Russian novels, seventies rock music, fashion magazines, travel mementos, or cultural or religious symbols—that can be used to establish a personal connection with the doctor.

Good details are dynamic; the best reps tailor their messages constantly according to their client's reaction. A friendly physician makes the rep's job easy, because the rep can use the “friendship” to request favors, in the form of prescriptions. Physicians who view the relationship as a straightforward goods-for-prescriptions exchange are dealt with in a businesslike manner. Skeptical doctors who favor evidence over charm are approached respectfully, supplied with reprints from the medical literature, and wooed as teachers. Physicians who refuse to see reps are detailed by proxy; their staff is dined and flattered in hopes that they will act as emissaries for a rep's messages. (See Table 1 for specific tactics used to manipulate physicians.)

**Table 1:**
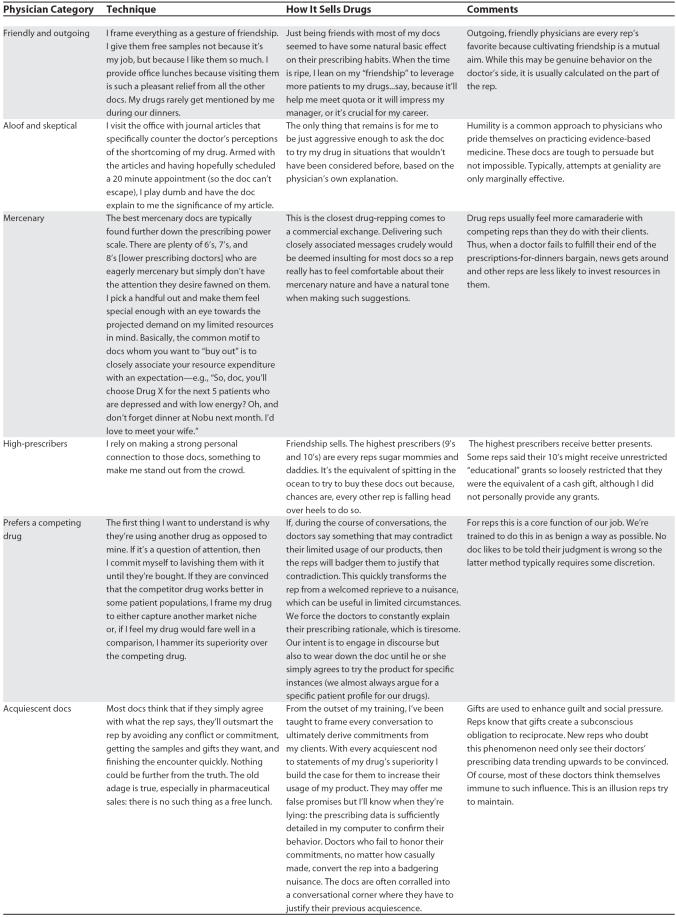
Tactics for Manipulating Physicians

**Table 1:**
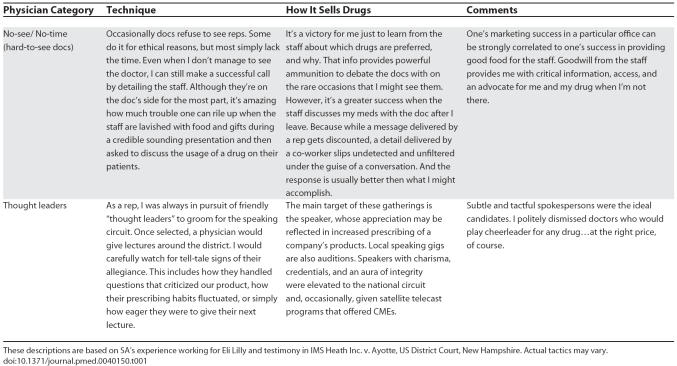
Continued

Gifts create both expectation and obligation. “The importance of developing loyalty through gifting cannot be overstated,” writes Michael Oldani, an anthropologist and former drug rep [26]. Pharmaceutical gifting, however, involves carefully calibrated generosity. Many prescribers receive pens, notepads, and coffee mugs, all items kept close at hand, ensuring that a targeted drug's name stays uppermost in a physician's subconscious mind. High prescribers receive higher-end presents, for example, silk ties or golf bags. As Oldani states, “The essence of pharmaceutical gifting…is ‘bribes that aren't considered bribes’” [1].

Reps also recruit and audition “thought leaders” (physicians respected by their peers) to groom for the speaking circuit. Physicians invited and paid by a rep to speak to their peers may express their gratitude in increased prescriptions (see Table 1). Anything that improves the relationship between the rep and the client usually leads to improved market share.

## Script Tracking

*An official job description for a pharmaceutical sales rep would read: Provide health-care professionals with product information, answer their questions on the use of products, and deliver product samples. An unofficial, and more accurate, description would have been: Change the prescribing habits of physicians.*

—James Reidy [4]

Pharmaceutical companies monitor the return on investment of detailing—and all promotional efforts—by prescription tracking. Information distribution companies, also called health information organizations (including IMS Health, Dendrite, Verispan, and Wolters Kluwer), purchase prescription records from pharmacies. The majority of pharmacies sell these records; IMS Health, the largest information distribution company, procures records on about 70% of prescriptions filled in community pharmacies. Patient names are not included, and physicians may be identified only by state license number, Drug Enforcement Administration number, or a pharmacy-specific identifier [5]. Data that identify physicians only by numbers are linked to physician names through licensing agreements with the American Medical Association (AMA), which maintains the Physician Masterfile, a database containing demographic information on all US. physicians (living or dead, member or non-member, licensed or non-licensed). In 2005, database product sales, including an unknown amount from licensing Masterfile information, provided more than $44 million to the AMA [5].

Pharmaceutical companies are the primary customers for prescribing data, which are used both to identify “high-prescribers” and to track the effects of promotion. Physicians are ranked on a scale from one to ten based on how many prescriptions they write. Reps lavish high-prescribers with attention, gifts, and unrestricted “educational” grants (Table 1). Cardiologists and other specialists write relatively few prescriptions, but are targeted because specialist prescriptions are perpetuated for years by primary care physicians, thus affecting market share.

Reps use prescribing data to see how many of a physician's patients receive specific drugs, how many prescriptions the physician writes for targeted and competing drugs, and how a physician's prescribing habits change over time. One training guide states that an “individual market share report for each physician…pinpoints a prescriber's current habits” and is “used to identify which products are currently in favor with the physician in order to develop a strategy to change those prescriptions into Merck prescriptions” [6].

A *Pharmaceutical Executive* article states, “A physician's prescribing value is a function of the opportunity to prescribe, plus his or her attitude toward prescribing, along with outside influences. By building these multiple dimensions into physicians' profiles, it is possible to understand the ‘why’ behind the ‘what’ and ‘how’ of their behavior.” [7] To this end, some companies combine data sources. For example, Medical Marketing Service “enhances the AMA Masterfile with non-AMA data from a variety of sources to not only include demographic selections, but also behavioral and psychographic selections that help you to better target your perfect prospects” [8].

The goal of this demographic slicing and dicing is to identify physicians who are most susceptible to marketing efforts. One industry article suggests categorizing physicians as “hidden gems”: “Initially considered ‘low value’ because they are low prescribers, these physicians can change their prescribing habits after targeted, effective marketing.” “Growers” are “Physicians who are early adopters of a brand. Pharmaceutical companies employ retention strategies to continue to reinforce their growth behavior.” Physicians are considered “low value” “due to low category share and prescribing level” [9].

In an interview with *Pharmaceutical Representative*, Fred Marshall, president of Quantum Learning, explained, “… One type might be called ‘the spreader’ who uses a little bit of everybody's product. The second type might be a ‘loyalist’, who's very loyal to one particular product and uses it for most patient types. Another physician might be a ‘niche’ physician, who reserves our product only for a very narrowly defined patient type. And the idea in physician segmentation would be to have a different messaging strategy for each of those physician segments ” [10].

In *Pharmaceutical Executive*, Ron Brand of IMS Consulting writes “…integrated segmentation analyzes individual prescribing behaviors, demographics, and psychographics (attitudes, beliefs, and values) to fine-tune sales targets. For a particular product, for example, one segment might consist of price-sensitive physicians, another might include doctors loyal to a given manufacturers brand, and a third may include those unfriendly towards reps” [11].

In recent years, physicians have become aware of—and dismayed by—script tracking. In July 2006, the AMA launched the Prescribing Data Restriction Program (see http://www.ama-assn.org/ama/pub/category/12054.html), which allows physicians the opportunity to withhold most prescribing information from reps and their supervisors (anyone above that level, however, has full access to all data). According to an article in *Pharmaceutical Executive*, “Reps and direct managers can view the physician's prescribing volume quantiled at the therapeutic class level” and can still view aggregated or segmented data including “categories into which the prescriber falls, such as an early-adopter of drugs, for example….” [12]. The pharmaceutical industry supports the Prescribing Data Restriction Program, which is seen as a less onerous alternative to, for example, state legislation passed in New Hampshire forbidding the sale of prescription data to commercial entities [13].

## The Value of Samples

The purpose of supplying drug samples is to gain entry into doctors' offices, and to habituate physicians to prescribing targeted drugs. Physicians appreciate samples, which can be used to start therapy immediately, test tolerance to a new drug, or reduce the total cost of a prescription. Even physicians who refuse to see drug reps usually want samples (these docs are denigrated as “sample-grabbers”). Patients like samples too; it's nice to get a little present from the doctor. Samples also double as unacknowledged gifts to physicians and their staff. The convenience of an in-house pharmacy increases loyalty to both the reps and the drugs they represent.

Some physicians use samples to provide drugs to indigent patients [14,15]. Using samples for an entire course of treatment is anathema to pharmaceutical companies because this “cannibalizes” sales. Among the aims of one industry sample-tracking program are to “reallocate samples to high-opportunity prescribers most receptive to sampling as a promotional vehicle” and “identify prescribers who were oversampled and take corrective action immediately” [16].

Studies consistently show that samples influence prescribing choices [14,15,17]. Reps provide samples only of the most promoted, usually most expensive, drugs, and patients given a sample for part of a course of treatment almost always receive a prescription for the same drug.

## Funding Friendship

*While it's the doctors' job to treat patients and not to justify their actions, it's my job to constantly sway the doctors. It's a job I'm paid and trained to do. Doctors are neither trained nor paid to negotiate. Most of the time they don't even realize that's what they're doing…*

—Shahram Ahari

Drug costs now account for 10.7% of health-care expenditures in the US [18]. In 2004, spending for prescription drugs was $188.5 billion, almost five times as much as what was spent in 1990 [19]. Between 1995 and 2005, the number of drug reps in the US increased from 38,000 to 100,000 [20], about one for every six physicians. The actual ratio is close to one drug rep per 2.5 targeted doctors [21], because not all physicians practice, and not all practicing physicians are detailed. Low-prescribers are ignored by drug reps.

Physicians view drug information provided by reps as a convenient, if not entirely reliable, educational service. An industry survey found that more than half of “high-prescribing” doctors cited drug reps as their main source of information about new drugs [22]. In another study, three quarters of 2,608 practicing physicians found information provided by reps “very useful” (15%) or “somewhat useful” (59%) [23]. However, only 9% agreed that the information was “very accurate”; 72% thought the information was “somewhat accurate”; and 14% said that it was “not very” or “not at all” accurate.

Whether or not physicians believe in the accuracy of information provided, detailing is extremely effective at changing prescribing behavior, which is why it is worth its substantial expense. The average annual income for a drug rep is $81,700, which includes $62,400 in base salary plus $19,300 in bonuses. The average cost of recruiting, hiring, and training a new rep is estimated to be $89,000 [24]. When expenses are added to income and training, pharmaceutical companies spend $150,000 annually per primary care sales representative and $330,000 per specialty sales representative [25]. An industry article states, “The pharmaceutical industry averages $31.9 million in annual sales spending per primary-care drug…Sales spending for specialty drugs that treat a narrowed population segment average $25.3 million per product across the industry.” [25]

## Conclusion

As one of us (SA) explained in testimony in the litigation over New Hampshire's new ban on the commercial sale of prescription data, the concept that reps provide necessary services to physicians and patients is a fiction. Pharmaceutical companies spend billions of dollars annually to ensure that physicians most susceptible to marketing prescribe the most expensive, most promoted drugs to the most people possible. The foundation of this influence is a sales force of 100,000 drug reps that provides rationed doses of samples, gifts, services, and flattery to a subset of physicians. If detailing were an educational service, it would be provided to all physicians, not just those who affect market share.

Physicians are susceptible to corporate influence because they are overworked, overwhelmed with information and paperwork, and feel underappreciated. Cheerful and charming, bearing food and gifts, drug reps provide respite and sympathy; they appreciate how hard doctor's lives are, and seem only to want to ease their burdens. But, as SA's New Hampshire testimony reflects, every word, every courtesy, every gift, and every piece of information provided is carefully crafted, not to assist doctors or patients, but to increase market share for targeted drugs (see Table 1). In the interests of patients, physicians must reject the false friendship provided by reps. Physicians must rely on information on drugs from unconflicted sources, and seek friends among those who are not paid to be friends.

## 

**Figure:**
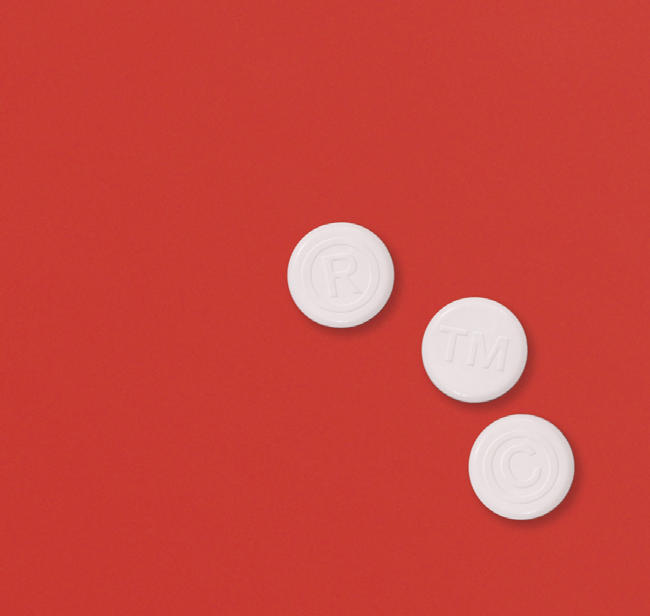
(Photo: “Bitter Pills?” by net_efekt, at http://www.flickr.com/photos/wheatfields/316337784/. Published under the Creative Commons Attribution License.)

**Figure:**
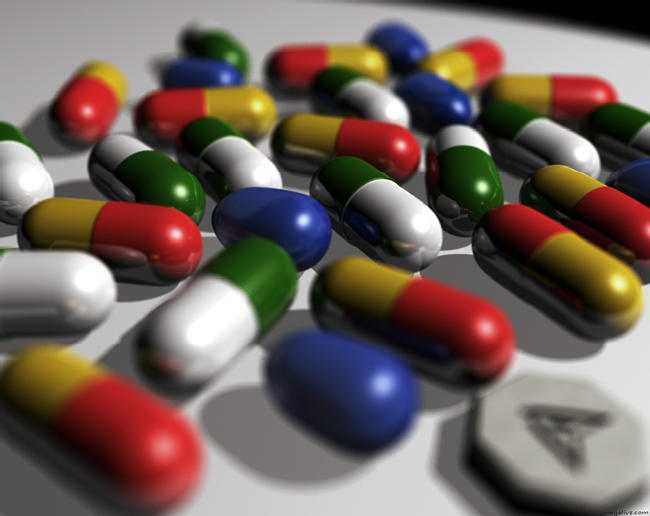
(Photo: “Pills” by Rodrigo Senna, at http://www.flickr.com/photos/negativz/74267002/. Published under the Creative Commons Attribution License.)

**Figure:**
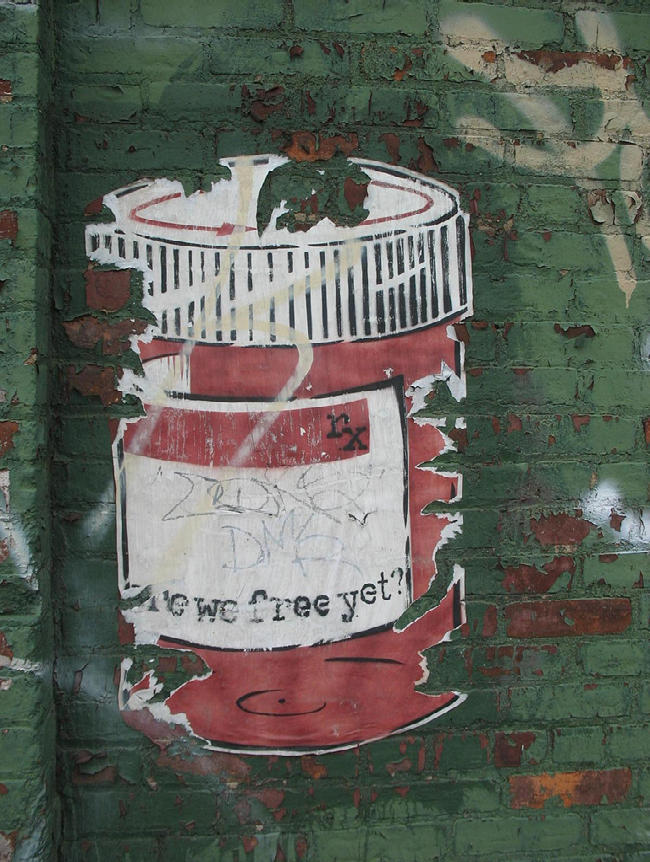
(Photo: “Pills” by Sugar Pond, at http://www.flickr.com/photos/sugarpond/236235191/. Published under the Creative Commons Attribution License.)

## Abbreviations

AMA: American Medical Association

## Note Added in Proof

Reference 26 is cited out of order in the article because it was added while the article was in proof.
